# Environmentally Controlled Microfluidic System Enabling Immune Cell Flow and Activation in an Endothelialised Skin‐On‐Chip

**DOI:** 10.1002/adhm.202400750

**Published:** 2024-10-06

**Authors:** Elisabetta Michielon, Matteo Boninsegna, Taco Waaijman, Dario Fassini, Sander W. Spiekstra, Jeremy Cramer, Pierre Gaudriault, János Kodolányi, Tanja D. de Gruijl, Antoni Homs‐Corbera, Susan Gibbs

**Affiliations:** ^1^ Department of Molecular Cell Biology and Immunology Amsterdam UMC Location Vrije Universiteit Amsterdam De Boelelaan 1117 Amsterdam 1081 HV The Netherlands; ^2^ Amsterdam institute for Infection and Immunity Amsterdam University Medical Center Vrije Universiteit Amsterdam The Netherlands; ^3^ Cancer Center Amsterdam Cancer Biology and Immunology Program Amsterdam UMC Vrije Universiteit Amsterdam 1081 HV The Netherlands; ^4^ Cherry Biotech SAS 14 Rue De La Beaune, Bâtiment A, 2ème Étage Montreuil 93100 France; ^5^ Department of Physics Bielefeld University Universitätsstr 25 33615 Bielefeld Germany; ^6^ Department of Dental Material Science Academic Centre for Dentistry Amsterdam (ACTA) University of Amsterdam and Vrije Universiteit Amsterdam 1081 LA The Netherlands; ^7^ Department of Medical Oncology Amsterdam UMC Location Vrije Universiteit Amsterdam Amsterdam 1081 HV The Netherlands; ^8^ Department of Oral Cell Biology, Academic Centre for Dentistry Amsterdam (ACTA) University of Amsterdam and Vrije Universiteit Amsterdam 1081 LA The Netherlands

**Keywords:** immune cell activation, immune cell flow, microfluidics, organ‐on‐chip, reconstructed human skin, skin‐on‐chip

## Abstract

Integration of reconstructed human skin (RhS) into organ‐on‐chip (OoC) platforms addresses current limitations imposed by static culturing. This innovation, however, is not without challenges. Microfluidic devices, while powerful, often encounter usability, robustness, and gas bubble issues that hinder large‐scale high‐throughput setups. This study aims to develop a novel re‐usable multi‐well microfluidic adaptor (MMA) with the objective to provide a flexible tool for biologists implementing complex 3D biological models (e.g., skin) while enabling simultaneous user control over temperature, medium flow, oxigen (O_2_), nitrogen (N_2_), and carbon dioxide (CO_2_) without the need for an incubator. The presented MMA device is designed to be compatible with standard, commercially available 6‐well multi‐well plates (6MWPs) and 12‐well transwells. This MMA‐6MWP setup is employed to generate a skin‐on‐chip (SoC). RhS viability is maintained under flow for three days and their morphology closely resembles that of native human skin. A proof‐of‐concept study demonstrates the system's potential in toxicology applications by combining endothelialised RhS with flowing immune cells. This dynamic setting activates the monocyte‐like MUTZ‐3 cells (CD83 and CD86 upregulation) upon topical exposure of RhS to a sensitizer, revealing the MMA‐6MWP's unique capabilities compared to static culturing, where such activation is absent.

## Graphical abstract

This study develops a reusable microfluidic multi‐well microfluidic adaptor (MMA) compatible with standard 6‐well plates and 12‐well transwells, providing user control over temperature, medium flow, and gases without an incubator. The microfluidic device maintains reconstructed human skin viability for three days in a skin‐on‐chip setup. A proof‐of‐concept toxicology study demonstrates immune cell activation, which is absent in static culturing.

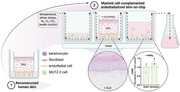

## 1 Introduction

Organ‐on‐chip (OoC) is a widely expanding approach that is expected to deliver superior human organotypic models for drug toxicity and efficacy testing, and is anticipated to be able to replace many animal experiments in the future.^[^ ^1^, ^2^, ^3^ ^]^ Today, various chip consoles and bioreactors are at different stages of development, ranging from experimental prototypes to commercially available models.^[^ ^4^, ^5^, ^6^ ^]^ However, limitations exist in terms of their ability to provide control of different microenvironments whilst at the same time being affordable by, e.g., being compatible with standard commercially available multi‐well plates (MWPs). Critical characteristics that need to be considered are the ability to control specific dissolved oxygen (O_2_) concentrations^[^ ^7^, ^8^, ^9^ ^]^ and pH conditions^[^ ^10^ ^]^ at physiological levels, as well as physical phenomena affecting the endothelial lining of the capillaries, such as wall shear stress (WSS).^[^ ^11^, ^12^, ^13^, ^14^ ^]^ Humidity and temperature are particularly important in the case of skin, which is exposed to ambient air in vivo. Additionally, many OoC designs incorporate organoids or micro‐vessels which create problems with sample size becoming very limiting for, e.g., multiplex and immunohistological analysis.

Skin is a challenging organ to incorporate into OoC platforms as air‐exposure to its surface is required for epidermal development, differentiation, and stratification, and nutrients are supplied via micro‐capillaries within the dermis. The outermost layer of the epidermis (*stratum corneum*) is thus subjected to ambient temperatures, whereas the dermis is exposed to core body temperatures. The skin is one of our largest organs forming an important barrier between the environment and our internal organs. Many drugs and consumer products applied topically as skin products or as a means of topical drug delivery (e.g., drug‐containing patches, creams, or ointments) may penetrate the skin and reach internal organs. Therefore, the skin barrier is relevant for systemic toxicity and drug bioavailability studies. Altogether, this makes it important to incorporate reconstructed human skin (RhS) models into OoC platforms.

Currently, many static air‐exposed RhS models are either commercially available, e.g., Phenion full‐thickness skin model^[^ ^15^ ^]^ (Henkel, Dusseldorf, Germany), T‐Skin human full‐thickness model^[^ ^16^ ^]^ (Episkin, Lyon, France), CELLnTEC full‐thickness skin model (CELLnTEC, Bern, Switzerland), and EpiDerm‐FT (Mattek, Ashland, USA), or used as complex three‐dimensional (3D) in‐house research models.^[^ ^17^, ^18^, ^19^, ^20^, ^21^, ^22^, ^23^ ^]^ The quality of these RhS is already to the level at which they are accepted by regulatory bodies as valid animal replacements. For example, reconstructed human epidermis (RhE) is regularly used to test skin corrosion, irritation, and dermal absorption of compounds according to the OECD Test Guidelines number 431^[^ ^24^ ^]^ and 439.^[^ ^25^ ^]^ Full‐thickness RhS models consisting of different combinations of epidermal cells (keratinocytes and melanocytes), immune cells (e.g., Langerhans cells (LCs)), and stromal cells (a combination of dermal fibroblasts, adipose stromal cells, endothelial cells (ECs), and dermal papilla cells) have been described.^[^ ^19^, ^21^, ^23^, ^26^, ^27^, ^28^, ^29^, ^30^, ^31^, ^32^, ^33^, ^34^ ^]^ Reconstructed skin barrier competency, although not to the level of that found in healthy human skin, is still adequate to enable the use of RhS in dermal penetration studies.^[^ ^35^ ^]^ The challenge is now to incorporate these models into OoC while retaining their current quality to facilitate systemic toxicity and bioavailability studies, which can be further adapted to multi‐OoC platforms.^[^ ^2^, ^36^, ^37^ ^]^

A range of OoC and multi‐OoC models incorporating skin has been described using commercially available microfluidic platforms. Research from us and other groups has reported the culturing of ex vivo skin or RhS alone or in combination with other organs.^[^ ^37^, ^38^, ^39^, ^40^ ^]^ Maschmeyer et al. combined human skin biopsies with either human liver spheroids or human intestine, liver spheroids, and human proximal tubule epithelial cells.^[^ ^38^, ^39^ ^]^ These OoC and multi‐OoC use HUMIMIC‐chips (TissUse, Berlin, Germany), which enable physiologically relevant shear stress and tissue‐to‐fluid ratios, as well as immune cell flow and vascular perfusion. In addition, while the skin has yet to be incorporated in Quasi Vivo from Kirkstall Ltd (York, UK), this system is compatible with most standard 24‐well‐format inserts and transwells, similar to TissUse.^[^ ^41^ ^]^ The console Zoë from Emulate (Boston, USA) allows the automatization of culturing conditions (i.e., gas composition and flow rate) for simultaneous cell culture of up to 12 custom designed organ‐chips. While these microfluidic systems provide valid platforms for various OoC applications, they still require the provision of an incubator and/or do not allow the maintenance of pre‐existing protocols. The aim of this study was thus to develop a novel re‐usable multi‐well microfluidic adaptor (MMA) device with the objective to provide a flexible microfluidic tool for biologists implementing complex 3D biological models, e.g., skin. The presented system was designed to be compatible with standard, commercially available 6‐well MWPs (6MWPs) and 12‐well transwell inserts, and to enable simultaneous user control over temperature, shear stress, carbon dioxide (CO_2_), O_2_, and nitrogen (N_2_) without the need for an incubator. This setup thus illustrates both the versatility and cost‐effectiveness of the scalable system.

As proof‐of‐concept for this technology, we showcase an endothelialised skin‐on‐chip (SoC), where air‐exposure and ambient temperature are implemented via the epidermal side, and core body temperature and medium flow for nutrient supply are implemented via the EC barrier and dermal side. ECs were integrated to mimic the vasculature, which can be considered as the first organ that will be affected after penetration of a substance through the skin. ECs lining the blood vessels are also critically implicated in immune responses by recruiting immune cells into tissues and organs.^[^ ^42^, ^43^ ^]^ Myeloid dendritic cell (DC) precursors (acute myeloid leukemia‐derived MUTZ‐3 progenitor cells^[^ ^44^, ^45^ ^]^) were placed in a well reservoir with dynamic flow enabling them to passage beneath the EC‐RhS barrier and were then collected in adjacent wells, thus enabling easy harvesting and analysis. RhS viability and histology under dynamic flow were compared to static conditions. Furthermore, immune cell activation after topical exposure of the RhS to the contact sensitizer nickel sulfate (NiSO_4_) was investigated.

## 2 Results

### 2.1 Design of a Microfluidic Multi‐Well Adaptor

**Figure 1:**
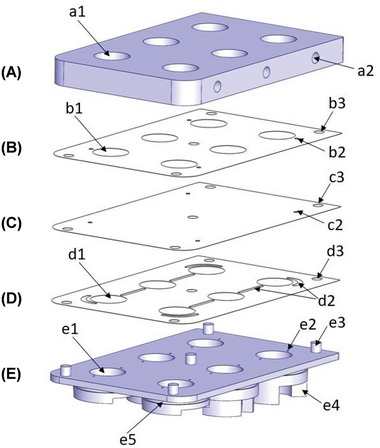
Schematic diagram of the microfluidic multi‐well adaptor (MMA) construct. A) Main manifold connecting (a2) external fluidic routing tubes for medium perfusion and allowing light transmission through (a1) the traversing round apertures. B) Biocompatible double‐side adhesive tape layer (142 µm‐thick) with through holes allowing (b2) fluidic routing and (b1) light transmission from (A) to (C). C) High transparency and auto‐fluorescence‐free (188 µm‐thick) COP layer with through holes allowing fluidic routing (c2) from (B) to (D). D) Biocompatible double‐side adhesive tape layer (142 µm‐thick) with patterned microfluidic channels (500 µm‐wide) allowing fluidic routing to the manifold (E) and between different wells (d2). (D) contains through holes for light transmission (d1) from (C) to (E). E) Manifold with through holes (e2) and nozzles (e4) allowing fluidic routing from (A) to a standard 6‐well plate (6MWP). Part (E) presents optical apertures (e1) allowing the transmission of light from a microscope to the biological sample once routed through (A), (B), (C), and (D). Part (E) has also assembled toroidal O‐rings (e5) guaranteeing the sealing, while assembled to the 6MWP, of the overall structure to external factors, such as contamination or gas environment. All parts have some extra features (b3, c3, d3, e3) to allow alignment of the multiple layers and to ease the assembling of the MMA.

A device with microfluidic channels was specifically designed, developed, and constructed based on patented technologies (Cherry Biotech, US11643632B2, FR3094012B1, and EP3712244A1). The device consisting of a MMA was designed for the first time to be compatible with commercially available 6MWPs and to accommodate 12 mm transwell inserts (782727; Brand GMBH, Wertheim, Germany). The MMA was made of five parts (**Figure** **1**): two Computer Numerical Control (CNC) machined blocks (Figure 1A,E), two patterned 142 µm‐thick biocompatible double‐side adhesive tape layers (ARseal 90880; Adhesives Research Ireland Ltd, Limerick, Ireland) (Figure 1B,D), and one high transparency and auto‐fluorescence‐free cycloolefin polymer (COP) 188‐µm‐thick layer (Zeonor 1420R; Zeon, Tokyo, Japan) (Figure 1C).

The design of the machined blocks was performed using SolidWorks v2017 (SolidWorks, Waltham, USA) and milled externally (ZEG‐MED Manufacturing Engineering Design, Ropczyce, Poland) using polycarbonate (PC). The first block, the main manifold (Figure 1A), was conceived to have six cylindrical apertures (a1 in Figure 1A) with a diameter of 17 mm to permit light transmission from a microscope. Lateral connectors (a2 in Figure 1A) were machined to have a UNF 1/4‐28 thread allowing the attachment of fluidic tubes ending with the complementing male connector. L‐shaped 1‐mm‐diameter channels were milled in this 6‐mm‐thick block to connect each of these lateral connectors (a2 in Figure 1A) to the microchannels (d2 in Figure 1D) in the successive parts of the device. This block was attached to the successive COP layer (Figure 1C) by means of one of the two double‐sided adhesive tapes (Figure 1B). The COP layer guarantees the structure sealing while maintaining the required properties to be able to image a sample within the 6MWP using a standard microscopic imaging setup. The second adhesive tape (Figure 1D) was patterned to define the 500 µm‐wide microfluidic channels (d2 in Figure 1D) connecting the first PC block to the second one through specific through holes in the COP and previous adhesive tape layers (Figure 1E). This second block defines a manifold also with six apertures (e1 in Figure 1E) and with fluidic 1‐mm‐diameter through holes (e2 in Figure 1E) connecting to the exterior and eventually to the 6MWP, when assembled to it, through a nozzle structure (e4 in Figure 1E). This manifold was completed with toroidal rubber O‐rings (e5 in Figure 1E) placed at the top of the nozzles guaranteeing sealing and isolation, when the MMA is assembled to a 6MWP, from external factors (e.g., contamination or uncontrolled gas environments). Adhesive tapes were patterned using a CE6000‐40 cutting plotter (Graphtec Corporation, Yokohama, Japan) and included six cylindrical apertures (b1 and d1 in Figure 1B,D, respectively) designed to be coincident with the corresponding ones in the machined PC blocks (a1 and e1 in Figure 1A,E, respectively). All parts were aligned using features specifically patterned for this purpose (b3, c3, d3, and e3 in Figure 1B,C,D,E, respectively).

### 2.2 Assembly of the MMA‐6MWP System

**Figure 2:**
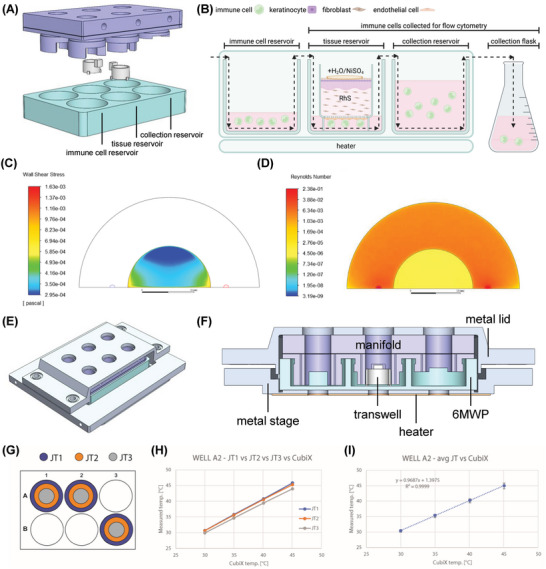
Assembly of the complete skin‐on‐chip (SoC) microfluidic device and flow characterization during perfusion. A) Expanded view of the MMA‐6MWP assembly including the MMA, the transwell cell culture insert, containing the RhS, and the 6MWP. B) Complete internal fluidic sealed structure of the myeloid cell‐complemented SoC model. The MMA connects three wells in a series. The direction of flow is indicated with dashed arrows. It is designed to maintain a very low volume of media in the entering well of the 6MWP holding circulating immune cells (“immune cell reservoir”). The second well connected to the previous one contains the RhS (“tissue reservoir”) and is designed to allow the flowed medium to contact only the EC layer at the bottom of the transwell insert. The third well works as a medium collector (“collection reservoir”). Excess medium is collected into an Erlenmeyer flask (“collection flask”). At the end of each experiment, the RhS and the flowed media can be recovered by opening the assembly. Created with BioRender.com. C) Modelled WSS at the transwell membrane: when applying a flow of 150 µL min^−1^, WSS values range between 2.95 × 10^−4^ Pa and 1.63 × 10^−3^ Pa, lower than those reported for human blood vessels in literature. D) Modelled Reynolds number at 1 µm under the transwell membrane when applying a 150 µL min^−1^ flow. Values range between 3.85 × 10^−6^ and 2.36 × 10^−5^ (laminarity regime under membrane). E) Heating holder of the assembled SoC MMA‐6WMP. F) Cross‐section of the heating holder that shows the MMA‐6MWP‐holder ensemble. A custom‐made polyamide heater integrated into the base of an aluminum plate warms the MMA‐6MWP ensemble, which has been designed to be compatible with real‐time imaging using a Leica DMi8 Inverted stage. G) Temperature calibration of the system was carried out by placing temperature probes (JTs) in three coaxial regions of three different wells near to the transwell membrane. The results allowed to assess H) zonal and I) mean weighted temperature of the culture medium to guarantee appropriate calibration of the temperatures set by the controlling unit.

The complete microfluidic device, creating the core of this perfused microphysiological system (MPS), resulted from assembling the previously described MMA with a Costar 6MWP (3506; Corning Life Sciences, Corning, USA) (MMA‐6MWP system). The MMA microchannels and the associated nozzles through holes were designed to generate two perfusion lanes serially connecting three wells (Figure 1 and **2**). The sealed microchannel and well perfusion lanes were connected to external tubing through an inlet and an outlet accessible through the corresponding MMA lateral connectors. These standard connectors permitted medium perfusion through inlet tubing and excess medium collection through outlet tubing for each lane. The symmetric structure was conceived with different designs of the nozzles in each of the connected wells with different functions and with specific nozzle heights (Figure 2A). The first pair of nozzles, placed in the well closer to the inlet (“immune cell reservoir”), defines a fluid volume of 200 µL to hold the small sample of circulating immune cells (e.g., MUTZ‐3 cell line used in this study). The second pair of nozzles is designed to transfer the medium to the middle well (“tissue reservoir”) while simultaneously holding in place a 12‐well transwell (782727; Brand GMBH), the membrane of which is 2 mm over the well bottom. This allows the RhS model in the insert to keep an epithelium‐to‐air interface created by the air trapped on top of the nozzle structure, which also acts as a bubble trap. The medium flowing from the previous well after entering the inlet nozzle is perfused underneath the transwell membrane before flowing to the adjacent well. The third pair of nozzles, placed in the well closer to the outlet (“collection reservoir”), holds a fluid volume of 8.5 mL to allow medium collection at the end of the experiments for downstream analyses. Excess medium is collected into the “collection flask” via outlet tubing.

The assembled SoC microfluidic device was modeled using Ansys computational fluid dynamics (CFD) software during the design phase to accomplish laminar flow at the transwell membrane level. A fluent solver was selected for the computational experiments setting steady time‐configuration and laminar flow module with no‐slip condition at the wall surfaces. The 3D model was simplified to avoid the computationally expensive representation of air‐liquid interfaces. Dulbecco's Modified Eagle Medium (DMEM; Gibco, Grand Island, USA) (0% fetal bovine serum) at 37 °C was set as fluid (*η* = 7.31 × 10^−4^ kg ms^−1^, *ρ* = 1.000 kg m^−3^, where *η* is the dynamic viscosity and *ρ* is the density^[^ ^46^ ^]^). The partial differential equations (PDEs) derived from Navier‐Stokes theory were solved through the Semi‐Implicit Method for Pressure Linked Equations (SIMPLE) algorithm.^[^ ^47^ ^]^ For the finite element method (FEM) mesh, the maximum size of the elements was set to 200 µm and the orthogonal quality was 0.786 ± 0.126.

The Reynolds number and the WSS affecting the ECs at the transwell membrane were calculated from the modeled velocity profiles considering and inlet flow of 150 µL min^−1^. The results of those simulations are presented in Figure 2C,D. The regime of the flow at 1 µm under the membrane was determined to be laminar with Reynolds numbers ranging between 3.85 × 10^−^^6^ and 2.36 × 10^−5^. The WSS produced by this flow to the cells attached to the membrane was very low and estimated to be between 2.95 × 10^−3^ and 1.63 × 10^−2^ dyn cm^−2^. The effect of different WSS magnitudes was not the objective of this study.

### 2.3 Environmental Control of the MMA‐6MWP System

**Figure 3:**
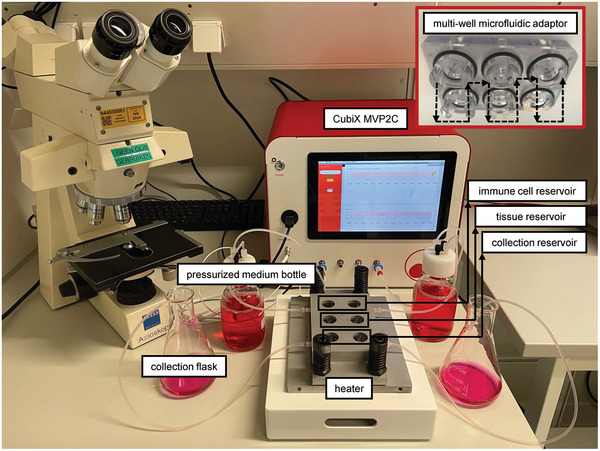
Complete MPS platform prototype (CubiX MVP2C) controlling the gaseous environment (percentages of CO_2_, N_2_, and O_2_), the perfusion (flow rate), and the heating (temperature) of the myeloid cell‐complemented SoC without the need for an external incubator. A detail of the constructed multi‐well microfluidic adaptor (MMA) is presented in the top‐right of the figure. Medium circulates from the pressurized medium bottle to the “collection flask” via the MMA as depicted by the black dashed arrows. The main components of the CubiX‐MMA‐6MWP‐heater system described in Figures 1 and 2 are labeled in white boxes.

A prototype developed by Cherry Biotech in 2019 named CubiX MVP2B was used for controlling the assembled microfluidic MMA‐6MWP device (**Figure** **3**). This controlling system provided stable temperatures, dissolved O_2_, and pH levels, as well as medium flow to the SoC. The platform was based on a Raspberry Pi3 Model B microprocessor and board (Raspberry Pi Ltd, Cambridge, UK). A tactile display (10.1″ 1280 × 800 HDMI Touchscreen; SunFounder, Shenzhen, China) and a Raspberry Pi High‐Precision AD/DA Expansion Board (Waveshare Electronics, Shenzhen, China) were added to allow full control with a specifically developed software. Integrated A/D converters, temperature controllers, and mass flow controlling drivers were written in Python programming language and a user interface was developed in JavaScript running a Raspbian operating system, Linux kernel 5.4.79, Devian v10 (Raspberry Foundation, Cambridge, UK). The software was conceived to easily allow the user to select the temperature, the percentages of O_2_, N_2_, and CO_2_ of the gas environment, and the flow of the perfused medium.

In order to control the temperature, a heating holder was conceived to heat the MMA‐6MWP ensemble while improving its sealing (Figure 2E,F). The heating holder consisted of two assembling main parts designed using SolidWorks v2017 and externally manufactured in aluminum (Protolabs, Shropshire, UK). The bottom part, i.e., the metal stage, was designed to fit and be compatible with real‐time imaging using a Leica DMi8 inverted stage (Leica, Wetzlar, Germany). A custom‐made polyamide heater (Huilong, Jiangsu, China) integrated in the base of this part warms the MMA‐6MWP‐holder ensemble. A lateral opening for a Pt100 sensor completed this metal stage which was filled with thermal paste (DC1; Bequiet, Glinde, Germany) and closed with the sensor. The sensor data were transmitted to the Raspberry Pi using a specific AD convertor (MAX31865; Maxim Integrated, Analog Devices, San Jose, USA). This signal was used to implement a Python adaptive real‐time closed‐loop algorithm on the Raspberry Pi to control the heating element and generate a user‐controlled temperature between 35 and 39 °C after accurate calibration. Temperature calibration of the system was carried out by placing simultaneously 9 JT Thermistor (SEMITEC, Tokyo, Japan) in three coaxial regions of three different wells in a 6MWP near to the transwell membrane of the RhS insert (Figure 2G). The results allowed us to assess zonal (Figure 2H) and mean weighted temperature (Figure 2I) of the culture medium using a 34972A LXI Data Acquisition/Switch Unit (Keysight, Santa Rosa, USA). This setup allowed to acquire the temperature at different experimental conditions and different 6MWP well positions, while also evaluating the temperature in the Pt100 integrated on the heating holder. These results were used to generate calibration curves and guarantee temperatures at the biological sample spot with an accuracy of ±0.1 °C by monitoring and adjusting the temperature at the Pt100 spot.

In order to perfectly control the gas in contact with the perfused medium and to simultaneously control its O_2_ and pH composition, and the desired medium flow, seven mass flow controlling units (MFC2242; Axetris AG, Kägiswil, Switzerland) were also integrated in the control device. Three of them were mounted to accurately control the independent flows of N_2_, O_2_, and CO_2_ through the Raspberry Pi to guarantee gas mixes containing percentages of these gases ranging from 0% to 100% in steps of 0.5% for each gas. The other four mass flow controlling units were used to regulate the gas outflows that also engender the required liquid flows through the MMA‐6MWP system. While the system was originally designed for controlling four perfused lanes, two were used in the final experiments described in this study. Seven pressure sensors (AP20; Fujikura, Tokyo, Japan) were integrated and monitored by using a TEC‐PVCHECK card (TecnoSens, Brescia, Italy), the Raspberry Pi High‐Precision AD/DA Expansion Board, and the Raspberry Pi during the overall control system operation.

In the system, the gas environment provided by the mixer is bubbled through the sealed medium bottles, which are connected to the inlet of the MMA‐6MWP ensemble (Figure 3), generating pressure and guaranteeing a stable pre‐set medium flow. This mechanism is described in Cherry Biotech patents US11643632B2, FR3094012B1, and EP3712244A1. Required dissolved O_2_ and pH levels are guaranteed through a repeatability of 0.15% on environment gas composition between experiments. Medium flow rate was ±3 µL min^−1^. The whole system does not require any external incubator. The full system while running an experiment is displayed in Figure 3, where a detail of the manufactured MMA can also be seen in the top‐right corner.

### 2.4 Establishment of a Skin‐On‐Chip Model

**Figure 4:**
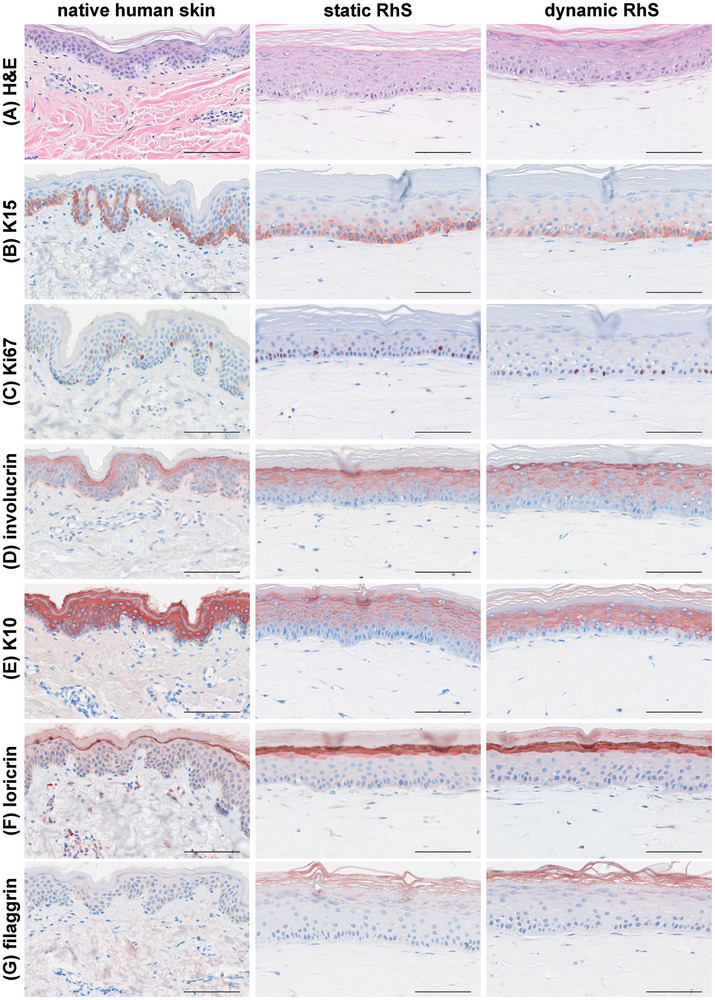
Histological analysis of reconstructed human skin (RhS) in static and dynamic conditions resembles native human skin. Histology of normal human skin and RhS sections by A) hematoxylin and eosin (H&E) staining and immunohistochemical stainings using antibodies directed against B) the basal epidermal layer marker keratin 15 (K15), C) the proliferation marker Ki67, the intermediate epidermal differentiation markers D) involucrin and E) K10, F) the late differentiation marker loricrin, and G) the cornified envelope protein filaggrin. Representative pictures of stained 5‐µm‐thick paraffin‐embedded RhS sections derived from *n* = 3 independent experiments performed in triplicate for static and duplicate for dynamic conditions are shown. Scale bar = 100 µm.

In order to determine whether RhS could be maintained under dynamic flow in the MMA assembled with a 6MWP (MMA‐6MWP), RhS was first integrated for three days for an extensive quality and stability test. A schematic overview of how RhS was constructed prior to their incorporation in the dynamic setting is displayed in Figure S1 (Supporting Information). Consistent with previous publications,^[^ ^20^, ^48^ ^]^ prior to its incorporation into the MMA‐6MWP system, the static RhS consisted of a stratified and differentiated epidermis on a fibroblast‐populated dermal compartment (**Figure** **4**A). In line with native human skin, staining for markers of different stages of epidermal differentiation revealed early (keratin 15 (K15)) (Figure 4B), intermediate (K10 and involucrin) (Figure 4D,E), and late (loricrin and filaggrin) (Figure 4F,G) epidermal differentiation. Proliferative keratinocytes, identified as Ki67^+^ cells, were found in the basal layer of the epidermis (Figure 4C). The incorporation of RhS in the MMA‐6MWP system for three days did not affect this RhS morphology, as expression levels of the above‐mentioned markers were comparable between the dynamic RhS and their static counterparts (Figure 4).

To further investigate whether a stable RhS culture under dynamic flow had been achieved, RhS viability was investigated by MTT assay, which assesses metabolic activity, and lactate dehydrogenase (LDH) release assay, which assesses LDH leaking from damaged cell membranes, and compared to that of static controls (Figure S2, Supporting Information). As for the MTT test, no difference in absorbance measurements could be observed between the two culturing conditions (Figure S2A, Supporting Information). To assess RhS viability as a means of LDH release, released LDH levels from RhS cultures were normalized to the mean LDH secretion from two dead static RhS cultures (0% viability). RhS were viable both in static and dynamic conditions, as their viability ranged between 97% and 100% (Figure S2B, Supporting Information). A slight decrease, albeit not significant, could be observed in the static controls over the three day period, likely due to the accumulation of LDH over time (medium was not refreshed). A stable low LDH release was found for the dynamic RhS up to 42 h (latest checked time point) after integration into the MMA‐6MWP system.

Overall, these results show that RhS are stable for at least three days in dynamic conditions and that the continuous flow affected neither RhS morphology and biological features nor tissue viability.

### 2.5 Establishment of a Myeloid Cell‐Complemented Endothelialised Skin‐On‐Chip Model

In order to mimic the EC barrier, ECs were seeded onto the underside of the RhS‐containing transwell. To introduce a flowing immune cell component, MUTZ‐3 monocyte‐like cells were placed in the “immune cell reservoir” (Figure 2B). Cells flowed to the adjacent well (“tissue reservoir”) containing the RhS and were collected into the third well (“collection reservoir”). Extra medium volume was collected in the “collection flask”. As a proof‐of‐concept study, for immune cell activation, air‐exposed endothelialised RhS were topically exposed to either the contact sensitizer NiSO_4_ or its vehicle water (H_2_O) with MUTZ‐3 cells flowing underneath each transwell for 24 h.

**Figure 5:**
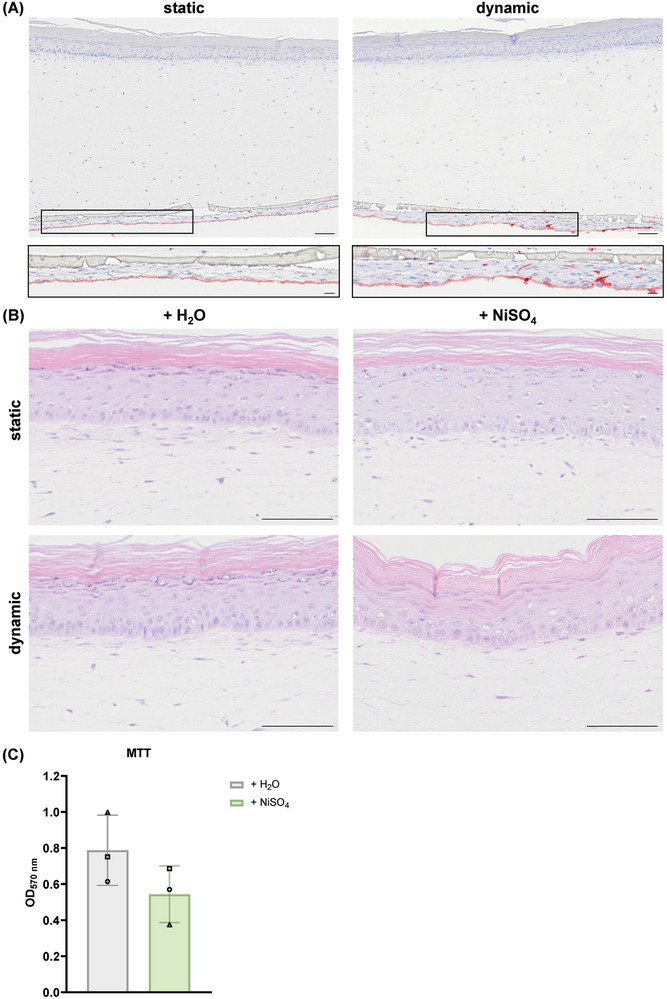
Exposure of NiSO_4_ marginally affects endothelialised RhS morphology and viability in static and dynamic conditions. A) CD31 staining to visualize the ECs underneath the RhS in static or dynamic conditions. Magnifications of CD31^+^ cells are shown in black rectangles. Scale bar = 100 µm. B) Hematoxylin and eosin (H&E) staining of endothelialised RhS exposed to either H_2_O or NiSO_4_ (190 mm) for 24 h in static or dynamic conditions (scale bar = 100 µm) and C) MTT assay of endothelialised RhS exposed to either H_2_O or NiSO_4_ (190 mm) for 24 h in static conditions. In these experiments, MUTZ‐3 cells were co‐cultured with the endothelialised RhS models. Data from *n* = 3 independent experiments are shown as mean ± SD. Each experiment is represented by a different symbol.

Staining for CD31 identified the presence of ECs underneath the RhS both in static and dynamic conditions (**Figure** **5**A), confirming the suitability of the MMA‐6MWP system to culture complex 3D models. A thin fibroblast layer can be observed between the transwell membrane and the ECs. This is most likely due to the leakage of fibroblast‐containing gel via the 8 µm‐pores of the transwell during the casting of the reconstructed dermis.

It was first investigated via inductively‐coupled plasma mass spectrometry (ICP‐MS) how much nickel penetrated via the static endothelialised RhS into the culture supernatant upon NiSO_4_ topical application (*n* = 1 with two intra‐experiment replicates). A negligible amount of nickel was detected in the supernatant from RhS exposed to H_2_O (mean ± SD = 3.8 ± 0.3 parts per billion (ppb)), while nickel concentration in the culture supernatant of RhS exposed to NiSO_4_ ranged from 7 to 2000 ppb (Figure S3, Supporting Information). Of note, nickel concentration in the NiSO_4_ solution used for exposure was measured to be 10.6 × 10^6^ ppb, indicating that only a very low amount of the topically applied nickel diffused through the RhS into the culture supernatant. Nickel ions were not measured in the dynamic setting as NiSO_4_ diffusing through the RhS into the culture medium would be diluted in 216 mL over the span of 24 h due to the unidirectional flow of the MMA‐6MPW system. Furthermore, it is currently unknown whether nickel binds to the materials used to make the microfluidic device. This might also account for the variation that was observed regarding MUTZ‐3 activation.

Next, RhS viability was assessed upon NiSO_4_ exposure, as topical application of high concentrations of NiSO_4_ can lead to mild skin toxicity.^[^ ^49^ ^]^ In one out of the three performed static experiments, exposure to NiSO_4_ increased vacuole formation in the epidermis, a sign of mild toxicity (Figure 5B). This corresponded to the lowest measured MTT value (triangle in + NiSO_4_ condition) in Figure 5C. Marginal tissue toxicity was seen in the other two static experiments, as shown in the negligible decrease in optical density at 570 nm (OD_570_) upon NiSO_4_ exposure (Figure 5C). In dynamic conditions, no 3 mm‐biopsies were taken for MTT to avoid sample limitation for staining purposes.

**Figure 6:**
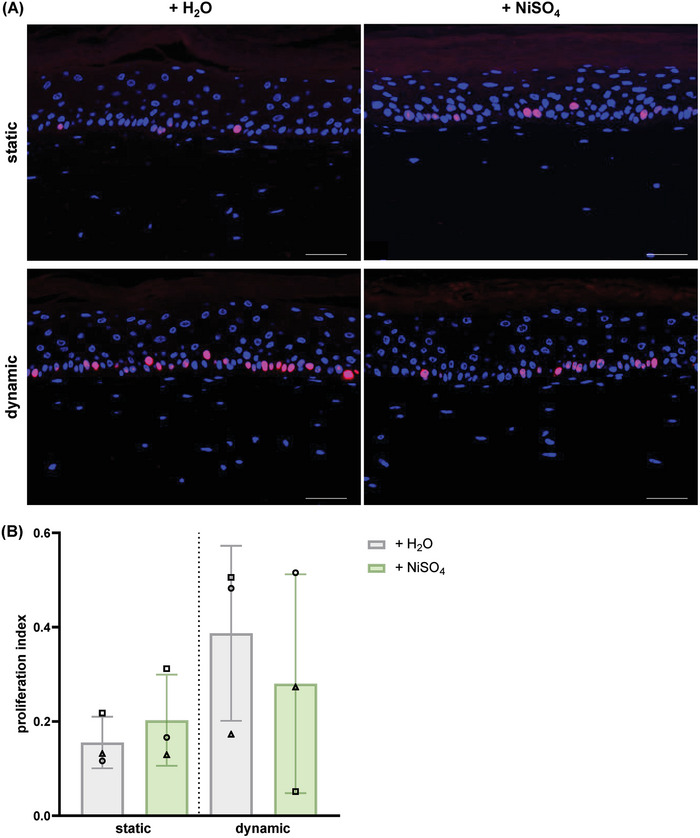
Dynamic flow affects epidermal cell proliferation. Proliferating epidermal cells were identified in endothelialised RhS exposed to either H_2_O or NiSO_4_ (190 mm) for 24 h in static or dynamic conditions A) via Ki67 (red) and DAPI (blue) immunofluorescent staining (scale bar = 50 µm). B) Ki67^+^ nuclei in the *stratum basale* were calculated and expressed as proliferation index. Data from *n* = 3 independent experiments are shown as mean ± SD. Each experiment is represented by a different symbol.

Upon investigating cell proliferation in RhS via immunofluorescent staining, an apparent increase in the number of proliferating cells, identified as Ki67^+^ cells, was observed in the basal layer of the epidermis in those RhS under dynamic conditions, compared to their static controls (**Figure** **6**A). When expressed as a proliferation index, RhS exposed to H_2_O showed this increase in all three experiments, although to different levels, while NiSO_4_‐exposed RhS showed an increase in two out of three experiments (Figure 6B).

**Figure 7:**
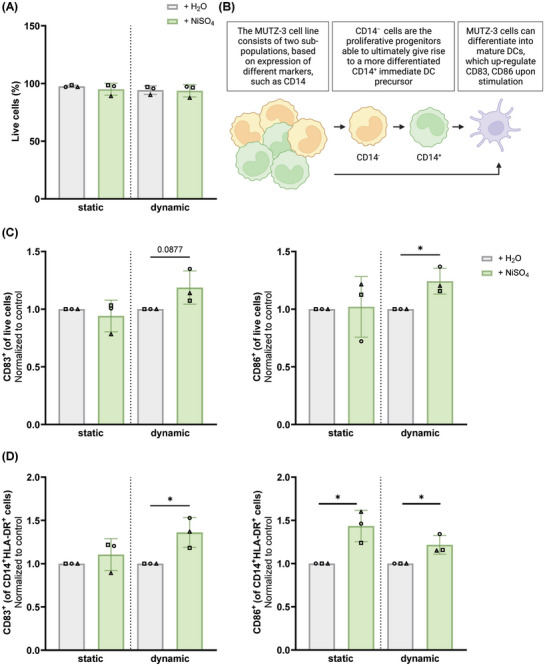
Exposure of endothelialised RhS to NiSO_4_ promotes the maturation of MUTZ‐3 cells in dynamic conditions. MUTZ‐3 cells were cultured in the presence of endothelialised RhS exposed to H_2_O (grey bars) or 190 mm NiSO_4_ (green bars) for 24 h in static or dynamic conditions and were assessed in terms of A) cell viability. Data from *n* = 3 independent experiments are shown as mean ± SD. Each experiment is represented by a different symbol. B) shows a simplified overview of the maturation process of the MUTZ‐3 cells. Created with BioRender.com and adapted from Santegoets et al.^[^ ^32^ ^]^ After co‐culture with the endothelialised RhS, expression of the surface markers CD83 and CD86 was measured within C) the live or D) the more differentiated CD14^+^HLA‐DR^+^ cell populations. CD83 and CD86 expression levels were normalized to the control + H_2_O (set as 1). Data from *n* = 3 independent experiments are shown as mean ± SD (^*^ *p* < 0.05; unpaired *t*‐test). Each experiment is represented by a different symbol.

It was next determined how flow affected MUTZ‐3 cell viability. The immune cells remained viable for 24 h and no differences in their viability could be observed between cells co‐cultured with the endothelialised RhS in static and dynamic conditions, regardless of RhS exposure to H_2_O or NiSO_4_ (**Figure** **7**A). This indicates that flow did not influence MUTZ‐3 cell viability and that the amount of nickel ions entering the culture supernatants (Figure S3, Supporting Information) was not cytotoxic to the MUTZ‐3 cells over the duration of the experiment.

**Figure 8:**
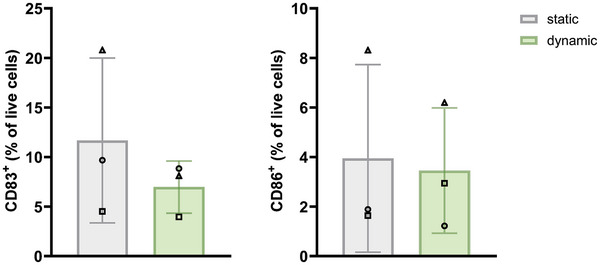
Dynamic flow does not influence MUTZ‐3 cell maturation in the presence of endothelialised RhS exposed to H_2_O. MUTZ‐3 cells were cultured in the presence of endothelialised RhS exposed to H_2_O in either static (grey bars) or dynamic conditions (green bars) for 24 h and were assessed in terms of expression of the surface markers CD83 and CD86. Data from *n* = 3 independent experiments are displayed as percentage (%) of live cells. Results are shown as mean ± SD. Each experiment is represented by a different symbol.

In vitro, the differentiation of MUTZ‐3 cells into maturing DCs involves the upregulation of the co‐stimulatory markers CD83 and CD86^[^ ^44^ ^]^ (Figure S4, Supporting Information). A simplified overview of MUTZ‐3 cell maturation is displayed in Figure 7B. To assess whether differentiation and maturation of MUTZ‐3 cells occurred upon contact with the NiSO_4_ diffusing through the endothelialised RhS model, the phenotype of cells collected from either the dynamic cultures (“tissue reservoir”, “collection reservoir”, and “collection flask” in Figure 2A,B) or their static controls was analyzed via flow cytometry (Figure 7C,D). Under static conditions, no differences in CD83 and CD86 levels were observed between H_2_O (CD83 expression range: 4.1%–22.8% and CD86 expression range: 1.4%–8.8%) and NiSO_4_ (CD83 expression range: 4.6%–16.7% and CD86 expression range: 1.3%–12.1%) conditions when MUTZ‐3 cells were placed directly underneath the topically exposed endothelialised RhS (static in Figure 7C). Of note, the presence of ECs underneath the RhS did not influence MUTZ‐3 maturation as, when they were omitted from the static culture, similar expression values of CD83 and CD86 were obtained as when they were present (Figure S5, Supporting Information). However, under dynamic flow, NiSO_4_ exposure of endothelialised RhS resulted in a trend for CD83 upregulation and in a significant increase in CD86 expression (CD83 expression range: 4.3%–12% and CD86 expression range: 1.7%–7.5%), compared to the H_2_O control (CD83 expression range: 4%–8.8% and CD86 expression range: 1.2%–6.2%) (Figure 7B). No differences in CD34 or CD54 expression levels were observed (Figure S6, Supporting Information). On the other hand, when considering the more differentiated CD14^+^HLA‐DR^+^ MUTZ‐3 cell population, CD86 upregulation was observed both in dynamic (+ H_2_O condition: CD83 expression range: 4.8%–7.3% and CD86 expression range: 1.9%–8.1%; + NiSO_4_ condition: CD83 expression range: 5.7%–11.2% and CD86 expression range: 2.2%–9.3%) as well as in static cultures (+ H_2_O condition: CD83 expression range: 3.5%–27.2% and CD86 expression range: 0.9%–9.9%; + NiSO_4_ condition: CD83 expression range: 5.5%–22% and CD86 expression range: 1.2%–16.6%) upon NiSO_4_ exposure (Figure 7D). Raw data are displayed in Figure S7 (Supporting Information). These results suggest that in static conditions immune cell activation only occurs in the more differentiated CD14^+^HLA‐DR^+^ population upon NiSO_4_ exposure, while the dynamic flow in combination with NiSO_4_ exposure has an activating effect also on the more early precursors. Of note, flow alone was insufficient to induce MUTZ‐3 cell maturation, as no CD83 or CD86 upregulation could be found under dynamic conditions in comparison to the static controls (**Figure** **8**).

Overall, these results indicate that flow might pre‐dispose MUTZ‐3 DC precursors to mature when in contact with a sensitizing chemical such as NiSO_4._

## 3 Discussion

SoC systems incorporating skin models of different degrees of complexity, ranging from skin cell monolayers to full‐thickness skin models (i.e., RhS or ex vivo skin), have been reported to circumvent the current limitations of static culturing conditions.^[^ ^37^, ^40^, ^50^, ^51^, ^52^, ^53^, ^54^, ^55^, ^56^, ^57^, ^58^, ^59^ ^]^ However, devices exploiting the power of microfluidics often present issues of usability, robustness, and gas bubble appearances, which impair their use in large‐scale high‐throughput setups.^[^ ^6^ ^]^ The aim of this study was thus to develop an environmentally controlled microfluidic device allowing simultaneous user control over temperature, shear stress, CO_2_, O_2_, and N_2_ without the need for an incubator, suitable for studying interactions between organs (e.g., full‐thickness transwell‐grown RhS) and immune cells under dynamic conditions. Here, we present a novel microfluidic model compatible with standard MWPs and transwells, hence permitting the preservation of current laboratory work pipelines and standard operating procedures, and thus facilitating usability and user acceptance. Of note, we were the first to convert a standard MWP with OoC technology (organ‐on‐well).

The suitability of this system to biological applications was tested and demonstrated via the generation of a myeloid cell‐complemented SoC. We reported RhS viability under continuous flow for up to three days, as shown by histology (i.e., normal epidermal stratification and differentiation), MTT test, and LDH assay. These features were comparable to the static RhS controls, indicating that the environment of the microfluidic system was compatible with the culturing of RhS.

In the field of SoC, maintenance of a dry, air‐exposed surface of RhS constitutes a major issue in culturing RhS on a chip, as the build‐up of humidity in the upper chamber of SoC platforms usually results in wet RhS, leading to poor *stratum corneum* morphology and barrier function. Pressure‐induced perfusion of medium to the top of the RhS constitutes another challenge that adds to this problem. In this study, with the presented microfluidic setup, we observed normal epidermal differentiation and *stratum corneum* generation in the microfluidic device, although we did encounter a small drop of condensation liquid on top of one RhS in one out of the three reported experiments (a total of six RhS models were used in dynamic conditions). We observed normal skin morphology and expression of epidermal differentiation and proliferation markers in the RhS under flow, in line with native human skin, and no differences were found between dynamic and static models. It has been shown that constructing RhS directly inside an OoC improves epidermal morphogenesis and differentiation, and enhances barrier function, compared to the static setup.^[^ ^53^, ^60^ ^]^ Here, we made the deliberate choice of generating mature full‐thickness RhS before their integration into the MMA‐6MWP since one of the main goals of this study was first to showcase the compatibility of the developed microfluidic device with pre‐established laboratory pipelines rather than improving RhS quality. With this approach, the MMA‐6MWP may also be compatible with RhS grown on standard 12‐well transwells in 6‐well plates and thus should also be compatible with commercially available RhS. This makes the microfluidic device easy to implement in many laboratories, or even contract research organizations (CROs) that do not have experience in skin tissue engineering and prefer to buy RhS from commercial suppliers. It also gives these laboratories the opportunity to easily combine RhS with immune cells under flow.

In the future, it would be of interest to assess whether morphology and functions of the epidermal layer may be improved by constructing and culturing the RhS directly in the dynamic setting for an extended period of time. Skin barrier functions might also be improved by introducing a controlled dry air flow in the upper chamber, which would allow for both the generation of a dry external environment (rather than humid) as well as the removal of the cornified layers (desquamation), thereby preventing the continuous thickening of the *stratum corneum* over time. This would eventually lead to a more physiological system and mimic native human skin exposure to ambient air. Nevertheless, viable and stable cultures could be maintained in flow conditions for up to three days, which is an adequate time interval for acute toxicity studies.

To further showcase the possible applications of the MMA‐6MWP system, we combined an endothelialised RhS with flowing immune cells in a 24 h toxicology proof‐of‐concept study, in which the contact sensitizer NiSO_4_ was used to topically expose the RhS. Previous publications from our research group have reported mild NiSO_4_‐derived toxicity on the reconstructed epidermis of RhS cultured in static conditions.^[^ ^49^, ^61^ ^]^ In line with this, we also observed a very mild toxic effect of NiSO_4_ on RhS viability both in static as well as dynamic culturing.

Interestingly, a trend toward increased epidermal cell proliferation, measured as proliferation index, in the *stratum basale* was observed in RhS under flow, compared to their static controls, regardless of their exposure to H_2_O or NiSO_4_. This suggests a potential role for dynamic flow in inducing epidermal cell proliferation.

While the trend in flow‐induced upregulation of proliferation could be observed in these one‐day experiments, no perceptible differences between dynamic versus static culturing were found when the experiment time was increased to three days. This might suggest a return to a constitutive proliferative population after a proliferative burst at day one, as reported by previous research.^[^ ^62^ ^]^

A slight increase in cell proliferation, albeit not significant, was also observed upon NiSO_4_ exposure in comparison to H_2_O, both in the dynamic and static groups. This is in line with research showing that treatment with NiSO_4_ (10 µg mL^−1^) of human primary keratinocytes and HaCaT cells induced DNA synthesis and cell proliferation.^[^ ^63^ ^]^

NiSO_4_ has also been previously reported to induce MUTZ‐3‐derived LC maturation and migration out of the epidermis and into the dermis.^[^ ^19^ ^]^ However, in this study, rather than the differentiated counterparts MUTZ‐3‐derived LCs or DCs, we selected MUTZ‐3 progenitor cells to mimic circulating monocytes in the peripheral bloodstream and showcase the system as a valuable tool for studying immune cell interactions with complex organs (i.e., skin). The EC layer was used to mimic human vasculature and did not induce immune cell activation, indicating that the observed MUTZ‐3 cell maturation was dependent on exposure to NiSO_4_, rather than the presence of this cell type.

Of note, in contrast to the static culturing condition, in which only the more differentiated CD14^+^HLA‐DR^+^ population was activated upon contact with the NiSO_4_ diffused through the RhS, the dynamic setting also prompted the maturation (i.e., upregulation of co‐stimulatory markers CD83 and CD86) of the more early MUTZ‐3 precursors. Indeed, this highlights the relevance of flow for toxicological studies to assess chemical‐induced immune cell activation, which may be missed in static culturing conditions.

While NiSO_4_ is able to directly initiate immune cell activation, it is well known that keratinocytes possess the ability to secrete tumor necrosis factor‐α (TNF‐α), interleukin‐1α (IL‐1α), and IL‐18 following skin sensitization.^[^ ^64^, ^65^, ^66^ ^]^ These cytokines generate cutaneous inflammation, which culminates in the regulation of LC migration and in the subsequent accumulation of DCs in the draining lymph nodes. These phenomena likely reflect the data presented in this paper. The low concentrations of nickel observed in the RhS‐derived culture supernatants, which was notably lower when compared to the NiSO_4_ solution concentration that was applied topically to the *stratum corneum*, could reflect the small fraction of nickel leaching from various sources (e.g., orthodontic appliances or jewelry) that is dermally absorbed. Therefore, MUTZ‐3 cell activation is likely the result of the combination of direct exposure to low concentrations of nickel and cytokines secreted by skin cells.

To our knowledge, this is the first reported microfluidic device allowing simultaneous user control over physiologically relevant parameters (temperature, shear stress, CO_2_, O_2_, and N_2_) without the need for an incubator. Specifically, the presence of a heating temperature regulator (heater) only underneath the 6MWP physiologically mimics core body temperature on the dermal side, whilst maintaining ambient temperature on the epidermal side. This precise temperature control could also be used to further investigate how temperature impacts epidermal morphogenesis, differentiation, and barrier functions^[^ ^27^ ^]^ in a dynamic setup. The materials in contact with the fluids and tissues have been selected to be completely small molecule absorbance‐free and impermeable to gases to preserve microenvironmental control.

The main limitation of this study was that, due to the relatively high flow rate (150 µL min^−1^) and the unilateral flow (i.e., culture medium had to be collected in a flask), large amounts of medium were used (≈216 mL/24 h). Therefore, we decided to culture the RhS in the MMA‐6MWP for three days maximum. To extend this culture time, flow rate should be reduced and this is the subject of our current research as well as follow‐up studies. However, considering that we aimed to flow immune cells beneath the RhS and collect them for phenotypic analysis to establish a myeloid cell‐complemented SoC model, culture time extension was not feasible in the current setup, as most immune cells had migrated into the “collection flask” after overnight incubation. Of note, our static RhS can be cultured for up to six weeks^[^ ^67^ ^]^ and we thus expect RhS to also be viable for this period of time in dynamic conditions, but such extended immune cell culture has yet to be reported in RhS co‐cultures.

Although the MMA‐6MWP system has been used here as a proof‐of‐concept study to generate a SoC, its compatibility to standard commercially available transwells makes it a customizable and scalable platform for other applications or for commercial models. Indeed, while we used 6MWPs and 12‐well transwell inserts, the system can be easily adapted to any other well format by using a different size MMA, allowing the end user to follow their standard operation procedures with little to no adjustments. The use of commercially available transwells also makes sample size not a limiting factor for the analysis pipeline, thus allowing the user to perform multiple downstream analyses (e.g., a combination of immunohistochemical analysis and omics studies) at the end of each experiment. Lastly, the MMA‐6MWP system has been developed to allow the interconnection of complementary organs in the future by linking the different tissue‐containing wells in a flexible way.

## 4 Conclusion

We report for the first time a novel microfluidic system combining standard MWPs with OoC technology (organ‐on‐well), which enables real‐time simultaneous user control over temperature, shear stress, CO_2_, O_2_, and N_2_ without the need for an incubator. These unique features, together with its versatility and ability to promote immune cell flow and interaction with complex organs (e.g., skin), make it a promising tool for advancing tissue culture research.

## 5 Experimental Section

### Cell Culture—Epidermal Cells, Dermal Fibroblasts, and Dermal Endothelial Cells

Human skin was obtained as surgical left‐over material after getting informed consent from healthy donors who underwent abdominal dermolipectomy and used in an anonymous fashion. Signed informed consent of patients and collection procedures were in compliance with the “Code of Conduct for Heath Research” as formulated by COREON (https://www.coreon.org/; Commissie Regelgeving Onderzoek) and with the approval of the local medical research ethics committee of the Amsterdam UMC.

Adipose tissue was removed from the skin and epidermis and dermis were separated by overnight incubation in dispase II (Roche, Basel, Switzerland) at 4 °C. Epidermal cells (keratinocytes and melanocytes) and dermal cells (dermal fibroblasts and dermal endothelial cells (ECs) were isolated by enzymatic treatment with trypsin and collagenase type II/dispase II, respectively. Epidermal cells and dermal fibroblasts were isolated and cultured as previously described^[^ ^48^, ^68^ ^]^ and were used up to the second or third passage, respectively, in all the experiments. Epidermal cells were cultured in Dulbecco's Modified Eagle Medium (DMEM; Gibco, Grand Island, USA) and Ham's F‐12 (Gibco) in a 3:1 ratio supplemented with 1% penicillin/streptomycin (P/S; Invitrogen, Paisley, UK), 1% UltroserG (UG; BioSepra S.A., Cergy‐Saint‐Christophe, France), 0.1 µm insulin (Sigma–Aldrich, St. Louis, USA), 1 µm hydrocortisone (Sigma–Aldrich), 1 µm isoproterenol (Sigma–Aldrich), and 2 ng mL^−1^ keratinocyte growth factor (KGF; Sigma–Aldrich) at 37 °C and 7.5% CO_2_. Dermal fibroblasts were cultured in DMEM with 1% P/S and 1% UG, referred to as fibroblast medium, at 37 °C and 5% CO_2_.

Dermal ECs were isolated, purified, and cultured as previously described.^[^ ^69^, ^70^ ^]^ Briefly, they were cultured on flasks pre‐coated with 1% gelatin (Sigma–Aldrich) in DMEM, 5% Fetal Clone III serum (Fisher Scientific, Loughborough, UK), and 1% P/S at 37 °C and 5% CO_2_ for ≈3–5 days until 70–80% confluence was reached. Purification of ECs was performed with a MidiMACS separator using microbeads against CD31 (Miltenyi Biotech, Bergisch Gladbach, Germany) following the manufacturer‘s protocol with three additional washing steps with phosphate‐buffered saline (PBS) after washing with medium. This step was repeated until a >99% pure population of CD31^+^CD90^−^ dermal ECs was obtained, as confirmed by flow cytometry. Purified ECs were then cultured on gelatin‐coated culture flasks in Medium 199 (M199; Lonza, Basel, Switzerland) containing 1% P/S, 2 mm l‐glutamine (Invitrogen), 10% heat‐inactivated new born calf serum (NBCS; Invitrogen), 10% human serum (Sanquin, Amsterdam, The Netherlands), 5 U mL^−1^ heparin (Pharmacy VUmc, Amsterdam, The Netherlands), and 3.75 µg mL^−1^ endothelial cell growth factor (ECGF) isolated from crude extract from bovine brain (Department of Physiology, Amsterdam UMC, Amsterdam, The Netherlands). This combined medium is referred to as human microvascular endothelial cell (hMVEC) medium and was supplemented with 2.5 ng mL^−1^ vascular endothelial growth factor (VEGF; PeproTech, Cranbury, USA) and 2.5 ng mL^−1^ basic fibroblast growth factor (bFGF; PeproTech) before use.

### Cell Culture—MUTZ‐3 Cell Line

The MUTZ‐3 progenitor cell line (Deutsche Sammlung von Mikroorganismen und Zellkulturen (DSMZ), Braunschweig, Germany) was maintained as previously described^[^ ^44^ ^]^ at 37 °C and 5% CO_2_. Cells were used until passage number 30 for all experiments.

### Construction of Reconstructed Human Skin

Resconstructed human skin (RhS) was constructed similarly to what previously described^[^ ^67^ ^]^ in 12‐well transwells (782727; Brand GMBH). A schematic overview of this process is displayed in Figure S6 (Supporting Information). Briefly, dermal equivalents were constructed by mixing fibroblasts (2.6 × 10^4^ cells/gel) with a rat‐tail collagen/fibrinogen (Diagnostica Stago SAS, Asnieres sur Seine, France) (1:1) solution. Each hydrogel contained 0.5 U mL^−1^ thrombin (Merck KGaA, Darmstadt, Germany) to allow for fibrin formation. After three days, epidermal cells were seeded onto the dermal equivalents at a density of 7 × 10^4^ cells/gel. After four days in submerged conditions, RhS were cultured at the air–liquid interface for at least seven days. During air‐exposure, the culture medium consisted of DMEM/Ham's F‐12 (3:1), 1% P/S, 0.2% UG, 1 µm isoproterenol, 1 µm hydrocortisone, 0.1 µm insulin, 2 ng mL KGF, 1 ng mL^−1^ epidermal growth factor (EGF; Sigma–Aldrich), 10 µm l‐serine (Sigma–Aldrich), 10 µm l‐carnitine (Sigma–Aldrich), 25 µm palmitic acid (Sigma–Aldrich), 7 µm arachidonic acid (Sigma–Aldrich), 15 µm linoleic acid (Sigma–Aldrich), 0.4 mm ascorbic acid (Sigma–Aldrich), and 1 µm vitamin E (Sigma–Aldrich), referred to as complete KCII, and was refreshed twice a week in static conditions.

### Integration of RhS into the MMA‐6MWP System

RhS were cultured at the airliquid interface for at least seven days prior to their integration into the MMA‐6MWP system. For each independent experiment, a RhS was placed in each “tissue reservoir” of the 6MWP and cultured for up to three days in dynamic conditions with a flow rate of 150 µL min^−1^. Samples of medium (complete KCII) were collected from the outlet tubing before reaching the “collection flask” each day, every hour, for about 7 h/day and stored at 4 °C (Figure 7). After 24 h, medium in the “collection flask” was sterilized over a 0.22 µm‐filter and placed back in the pressurized medium bottle (Figure 7) to be flowed again. Three RhS were used as static controls and incubated at 37 °C and 7.5% CO_2_ for three days per experiment. No medium refreshment was performed for these models and a sample of medium (100 µL) was collected at the end of each day and stored at 4 °C.

### Generation of Endothelialised RhS

In order to generate endothelialised RhS models, dermal ECs were seeded on the membrane underneath the transwell inserts one day after the fibroblast‐containing hydrogels were cast, as follows: 1) transwells were flipped upside down and membranes were coated with 50 µL of 1% gelatin for 30 min at 37 °C, 2) dermal ECs were seeded at a concentration of 6 × 10^4^ cells/membrane and were left to attach for 1.5 h at 37 °C and 5% CO_2_, 3) transwells were flipped back and submerged in medium consisting of 50% hMVEC medium supplemented with 2.5 ng mL^−1^ VEGF and 2.5 ng mL^−1^ bFGF and 50% fibroblast medium.

### Exposure of Endothelialised RhS to NiSO_4_ or H_2_O

After 14 days in air‐exposed conditions, endothelialised RhS were exposed either to the sensitizer nickel sulfate (NiSO_4_) (190 mm in water (H_2_O)) (1 067 270 100; Merck KGaA) or its vehicle H_2_O by impregnating gauze filters (Sefar Nitex, Heiden, Switzerland), as previously described.^[^ ^71^ ^]^ The impregnated gauze filters were applied topically onto the RhS, which were then placed in dynamic flow conditions for 24 h. Endothelialised RhS exposed to either NiSO_4_ or H_2_O cultured in static conditions at 37 °C and 7.5% CO_2_ were used as controls.

### Integration of MUTZ‐3 Cells and Endothelialised RhS into the MMA‐6MWP System

MUTZ‐3 cells were seeded in the “immune cell reservoir” of each lane of the 6MWP (as described in Figure 2A,B) at a concentration of 1 × 10^6^/well, while endothelialised RhS topically exposed to either H_2_O or 190 mm NiSO_4_ were placed in each “tissue reservoir”. This setup was placed in dynamic conditions with a flow rate of 150 µL min^−1^ and MUTZ‐3 cells flowed through to the adjacent wells of the 6MWP (Figure 2B). Complete KCII was used as medium. For static controls, 1 × 10^6^ MUTZ‐3 cells were seeded directly into the bottom of the 6MWP in 1.5 mL of complete KCII with the H_2_O‐ or NiSO_4_‐exposed endothelialised RhS‐transwell placed above. After 24 h, tissue sections were fixed in 4% formaldehyde overnight and prepared for immunohistological analysis, while MUTZ‐3 cells were collected from the static wells and from the “tissue reservoir”, “collection reservoir”, and “collection flask” of the MMA‐6MWP system (Figure 2B) for surface marker expression analysis by flow cytometry.

### Histology, Immunohistochemistry, and Immunofluorescent Staining

Tissue sections were fixed in 4% formaldehyde (VWR, Radnor, USA) overnight and prepared for immunohistological analysis. Formalin‐fixed paraffin‐embedded 5 µm‐thick skin and RhS tissue sections were used for morphological (hematoxylin and eosin staining, H&E), immunohistochemical, and/or immunofluorescent analysis of filaggrin (1:500, PRB‐417P; Covance, Emeryville, USA), involucrin (1:1000, clone SY5, NCL‐INV; Novocastra, Newcastle, UK), keratin 10 (K10) (1:100, clone DE‐K10, 11414; Progen, Heidelberg, Germany), keratin 15 (K15) (1:50, clone LHK15, Monx10690; Monosan, Uden, The Netherlands), Ki67 (1:50, clone MIB‐1, M7240; Dako, Glostrup, Denmark), loricrin (1:500, clone AF62, GC14643001; Covance), and CD31 (1:40, clone JC70A, M0823; Dako). Sections were deparaffinized and immersed in 0.01 m acetate buffer (pH 6.0) for filaggrin and Ki67 or in 10 mm Tris/1 mm EDTA buffer (pH 9.0) for CD31 for 15 min at 100 °C for antigen retrieval, followed by slowly cooling to room temperature. A 15 min incubation with pepsin was performed prior to antibody addition for K10 and K15. Sections were washed with PBS and stained for 1 h with the primary antibody. For immunohistochemistry, sections were then incubated with BrightVision plus Poly‐HRP‐Anti‐Mouse/Rabbit IgG (Immunologic, VWR International B.V., Breda, the Netherlands) and 3‐amino‐9‐ethylcarbazole (AEC; Sigma–Aldrich) substrate, and counterstained with hematoxylin. For immunofluorescent staining, sections were then incubated with a conjugated secondary antibody (goat anti‐mouse IgG AlexaFluor555, 1:200; Thermo Fisher Scientific, Waltham, USA) for 30 min and DAPI (1:5000, D3571; Invitrogen). Stained tissue sections were photographed using the VS200 slide scanner (Olympus, Tokyo, Japan). RhS tissue sections derived from three independent experiments were used for staining. The Ki67 signal in the AlexaFluor555 channel was detected if it was co‐localized with the nuclear marker DAPI in the basal layer of the reconstructed epidermis and analyzed with QuPath (v0.5.1).^[^ ^72^ ^]^ Proliferation index was calculated as follows:

(1) $$proliferation\;index=\frac{Ki67^{+}\;nuclei}{DAPI^{+}\;nuclei}$$

### MTT Assay

Metabolic activity was measured by means of the colorimetric conversion of thiazolyl blue tetrazolium bromide (MTT) into purple formazan crystals by NAD(P)H‐dependent oxidoreductases, as previously described.^[^ ^73^ ^]^ Briefly, 3 mm‐punch biopsies of RhS were incubated in 2 mg mL^−1^ MTT (Sigma–Aldrich) in the dark for 2 h at 37 °C. Biopsies were then transferred to an isopropanol and 0.04 M HCl (3:1) solution and incubated overnight in the dark at room temperature. The absorbance of the formazan dissolved in isopropanol was measured at 570 nm using the Mithras LB 940 microplate reader (Berthold Technologies, Bad Wildbad, Germany).

### LDH Assay

Viability of RhS was based on the measurement of lactate dehydrogenase (LDH) released from leaky or damaged cell membranes into culture supernatants, which were collected as described in the “Integration of RhS into the MMA‐6MWP System” section. LDH assay was performed using the cytotoxicity Detection KitPLUS (Roche), according to the manufacturer's instructions.

### ICP‐MS Measurements of NiSO_4_

Nickel concentrations in 24 h‐conditioned culture supernatants from static endothelialised RhS topically exposed to either H_2_O or NiSO_4_ were measured with a Nexion 1000 inductively‐coupled plasma mass spectrometer (ICP‐MS) (Perkin Elmer, Waltham, USA) in kinetic energy discrimination mode. The concentration of nickel in the NiSO_4_ solution used for exposure was also analyzed as a control. Before measurement, each sample was diluted with milli‐Q water (nickel content 3 ± 1 parts per trillion (ppt)) to decrease plasma load and to keep nickel signals comparable between samples.

### Flow Cytometry

MUTZ‐3 cells were collected and prepared for fluorescence‐activated cell sorting (FACS) analysis. Cells were washed with FACS buffer consisting of PBS supplemented with 0.1% bovine serum albumin (BSA) and 0.05% sodium azide (NaN_3_), and stained at 4 °C for 30 min with the following labelled antibodies: anti‐CD14‐PE‐Cy5 (BioLegend, San Diego, USA), anti‐CD34‐FITC (BD Biosciences, Franklin Lakes, USA), anti‐CD54‐PE (BD Biosciences), anti‐CD83‐APC‐Vio770 (Miltenyi Biotech), anti‐CD86‐APC (BD Biosciences), and anti‐HLA‐DR‐VioGreen (Miltenyi Biotech). Cells were then washed twice and resuspended in 500 µl of FACS buffer. Cells were stained with Sytoxblue (Thermo Fisher Scientific) before acquisition at Attune NxT Flow Cytometer (Thermo Fisher Scientific). Flow cytometry data were analyzed on FCS Express v7 (De Novo Software, Pasadena, USA).

### Statistical Analysis

Unless otherwise stated, all data are presented as mean ± standard deviation (SD) of three independent experiments, each using a different skin donor and a different MUTZ‐3 cell passage, when required. Statistical analysis was performed by means of an unpaired *t*‐test, as indicated in the Figure legends, using GraphPad Prism 10 software v10.2.0 (GraphPad Software Inc., La Jolla, USA). Differences were considered to be significant when *p* < 0.05.

## Conflict of Interest

Matteo Boninsegna, Dario Fassini, Pierre Gaudriault, Jeremy Cramer, and Antoni Homs‐Corbera have been or are currently affiliated with Cherry Biotech SAS, a French SME based in Montreuil commercializing instrumentation for accurate control and monitoring of in vitro systems aimed to provide reliable alternative methods to animal testing to the pharma and biotech industries. All other authors have no conflict of interest to declare.

## Author Contributions

E.M. and M.B. contributed equally to this work. E.M. performed formal analysis, investigation, validation, and visualized the project, wrote and prepared the original draft, and wrote, reviewed, and edited the final manuscript. M.B. performed investigation and validation, visualized the project, and wrote and prepared the original draft. T.W. performed investigation and validation, wrote, reviewed, and edited the final manuscript. D.F. performed investigation and validation. S.W.S. conceptualized the project, and performed formal analysis, investigation, and validation. J.C. acquired funds, wrote, reviewed, and edited the final manuscript. P.G. acquired funds, wrote, reviewed, and edited the final manuscript. J.K. performed investigation, and wrote, reviewed, and edited the final manuscript. T.D.dG. conceptualized the project, acquired funds, performed methodology, supervised the project, and wrote, reviewed, and edited the final manuscript. A.H.C. conceptualized the project, acquired funds, performed investigation, methodology, validation, and project administration, supervised the project, visualized the project, wrote and prepared the original draft, and wrote, reviewed, and edited the final manuscript. S.G. conceptualized the project, acquired funds, performed methodology and project administration, acquired funds, wrote and prepared the original draft, and wrote, reviewed, and edited the final manuscript.

## Supporting information



Supporting Information

## Data Availability

The data that support the findings of this study are available from the corresponding author upon reasonable request.
