# Supplementary material for: Environmentally Controlled Microfluidic System Enabling Immune Cell Flow and Activation in an Endothelialised Skin‐On‐Chip
Source: Adv Healthc Mater. 2024 Oct 6;13(29):2400750. doi: 10.1002/adhm.202400750 (PMC11582514; doi:10.1002/adhm.202400750)
Supplement: Supplementary file 1 — Supporting Information [file ADHM-13-0-s001.docx]

Supporting Information

**Figure S1. Schematic overview of the construction of endothelialised RhS and their integration into the 6MWP-MMA system.** Dermal equivalents were constructed by mixing fibroblasts with a rat-tail collagen/fibrinogen solution. The following day, dermal ECs were seeded on the membrane underneath the transwell inserts. After two days, epidermal cells were seeded onto the dermal equivalents. After four days in submerged conditions, RhS were cultured at the air-liquid interface for at least seven days before their integration into the microfluidic device (MMA-6MWP). Medium exchange was performed at each depicted step. Created with BioRender.com.


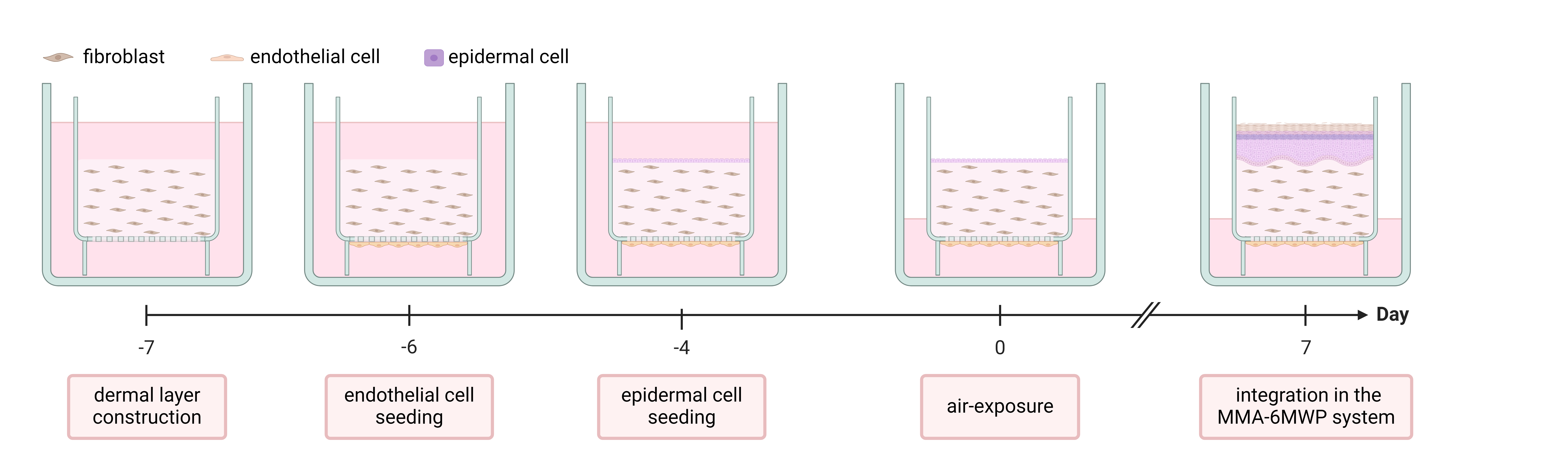


Figure S2. RhS are viable up to three days in dynamic conditions. Viability of RhS in the microfluidic device was compared to the static counterpart and assessed via (A) MTT assay and (B) LDH release in culture supernatants. LDH levels are normalized to the mean LDH secretion of two dead RhS cultures (positive controls set as 0% viability). Results are shown as mean  ± SD. N = 3 independent experiments performed in triplicate for static and duplicate for dynamic conditions. Each experiment is represented by a different symbol, when applicable.

**Figure S3. Amount of nickel diffusing into culture supernatants after endothelialised RhS were topically exposed to either H_2_O or NiSO_4_ (190 mM).** Nickel concentration in the NiSO_4_ solution used for topical exposure was 10.6×10^6^ ppb. Data from N = 1 experiment performed in duplicate are shown as mean ± standard error of the mean (SEM).

**Figure S4. Viability and activation of MUTZ-3 cells upon exposure to H_2_O or NiSO_4_ in 2D.** MUTZ-3 cells were exposed to either H_2_O or 800 μM NiSO_4_ for 24 hours in static conditions and were assessed in terms of **(A)** viability and expression of the surface markers CD83 and CD86 within **(B)** the live (H_2_O condition: CD83 expression range: 5.3% – 10.7% and CD86 expression range: 1.6% – 4.9%. NiSO_4_ condition: CD83 expression range: 5.5% – 13.1% and CD86 expression range: 2% – 12.5%) or **(C)** the CD14^+^HLA-DR^+^ cell populations (H_2_O condition: CD83 expression range: 10.4% – 15.4% and CD86 expression range: 1.6% – 6.3%. NiSO_4_ condition: CD83 expression range: 11.7% – 13.2% and CD86 expression range: 2.7% – 11.4%). Data from N = 3 independent experiments shown as mean ± SD. Each experiment is represented by a different symbol. Data regarding CD83 and CD86 expression were normalized to the control + H_2_O (set as 1).

**Figure S5. The presence of ECs underneath the RhS exposed to H_2_O does not influence MUTZ-3 cell maturation.** MUTZ-3 cells were cultured in static condition underneath RhS lined with ECs (+ ECs; green bars) or RhS without this EC-layer (- ECs; grey bars), which were exposed to the vehicle H_2_O for 24 hours. Maturation of MUTZ-3 cells was assessed in terms of expression of the surface markers CD83 and CD86. Data from N = 3 independent experiments are displayed as percentage (%) of live cells. Results are shown as mean ± SD. Each experiment is represented by a different symbol.

**Figure S6.** **Exposure of endothelialised RhS to NiSO_4_ does not influence CD34 or CD54 expression on MUTZ-3 cells.** MUTZ-3 cells were cultured in the presence of endothelialised RhS exposed to H_2_O (grey bars) or 190 mM NiSO_4_ (green bars) for 24 hours in static or dynamic conditions. Frequency percentage (%) of live cells expressing the surface marker CD34 and mean fluorescence intensity (MFI) of CD45 in the live cell population. Data from N  = 3 independent experiments shown as mean ± SD. Each experiment is represented by a different symbol.

**Figure S7.** **CD83 and CD86 expression on MUTZ-3 cells after co-culture with endothelialised RhS exposed to H_2_O or NiSO_4_ in static or dynamic conditions.** MUTZ-3 cells were cultured in the presence of endothelialised RhS exposed to H_2_O (grey bars) or 190 mM NiSO_4_ (green bars) for 24 hours in static or dynamic conditions. After co-culture with the endothelialised RhS, expression of the surface markers CD83 and CD86 was measured within **(A)** the live or **(B)** the more differentiated CD14^+^HLA-DR^+^ cell populations. Data from N  = 3 independent experiments shown as mean ± SD. Each experiment is represented by a different symbol.
